# Evaluation of the Transition from Holmium:YAG to Pulsed Thulium:YAG for Laser Endoscopic Enucleation of the Prostate and the Effect on Procedural Performance

**DOI:** 10.1016/j.euros.2026.01.010

**Published:** 2026-02-13

**Authors:** Maximilian Glienke, Maximilian Ferry von Bargen, Arif Özkan, Martin Schönthaler, Konrad Wilhelm, Christian Gratzke, Arkadiusz Miernik

**Affiliations:** Department of Urology, University of Freiburg Medical Center, Freiburg, Germany

**Keywords:** Benign prostatic hyperplasia, Pulsed thulium laser, Holmium laser, Laser enucleation of the prostate, Surgical transition

## Abstract

**Background and objective:**

Endoscopic enucleation of the prostate (EEP) using holmium:YAG laser (HoLEP) is widely regarded as the surgical gold standard for surgical management of benign prostatic hyperplasia. EEP with a pulsed thulium:YAG laser (ThuLEP) has emerged as an alternative with distinct physical properties that may influence surgical performance. However, evidence on the real-world impact of switching between laser platforms remains limited.

**Methods:**

In this retrospective single-center study, we analyzed 2688 consecutive EEP procedures performed between 2015 and 2025. Three high-volume surgeons transitioned from HoLEP (*n* = 1516) to ThuLEP (*n* = 1172) without retraining. Baseline characteristics, complication rates, and efficiency metrics were compared. Segmented regression analysis was used to evaluate temporal changes in performance across the laser transition.

**Key findings and limitations:**

Baseline parameters were comparable between the HoLEP and ThuLEP groups. ThuLEP was associated with significantly lower incidence of postoperative urinary retention (5.6% vs 9.3%; *p* = 0.0005) and a lower need for transurethral coagulation (3.0% vs 4.6%; *p* = 0.0471). EEP efficiency was higher with ThuLEP (0.93 ± 0.49 vs 1.64 ± 1.03 g/min; *p* < 0.001), accompanied by higher energy consumption (898.2 ± 692.1 vs 1,051.7 ± 1,003.3 J/g; *p* < 0.001). Segmented regression revealed an initial rise in efficiency after the transition, followed by mild performance fluctuations, which indicates a short adaptation phase.

**Conclusions and clinical implications:**

A transition from HoLEP to ThuLEP with a pulsed thulium:YAG laser is safe and feasible in experienced hands. ThuLEP was associated with higher enucleation efficiency and a statistically significant improvement in hemostatic control, without an increase in complication rates. These findings highlight the procedural adaptability of laser EEP techniques and support the clinical viability of platform switching in high-volume settings.

**Patient summary:**

We looked at outcomes in >2600 patients who underwent prostate surgery using two different laser systems. After switching from a holmium laser to a thulium laser, surgeons performed procedures more efficiently and with fewer cases of urinary retention and bleeding. This suggests that experienced surgeons can safely switch to newer laser systems without compromising patient care.

## Take Home Message

A transition from holmium laser to pulsed thulium laser for enucleation of the prostate is safe and feasible for experienced surgeons. Thulium:YAG laser improves the enucleation efficiency and hemostasis without increasing complication rates. These results support use of pulsed Tm:YAG laser as a viable alternative in high-volume clinical settings.

## 1 Introduction

Benign prostatic hyperplasia (BPH) is a prevalent age-related condition among men and represents one of the primary contributors to lower urinary tract symptoms (LUTS) [1]. Surgical intervention becomes necessary in patients with an inadequate response to or failure of medical therapy, and in those presenting with other clinical indications, such as refractory urinary retention or obstruction-related complications. Among the options available, laser enucleation of the prostate (LEP) has emerged as the gold standard for surgical management of benign prostatic obstruction, and offers excellent long-term outcomes, minimal bleeding, and short hospital stays [2], [3].

However, LEP involves a notable learning curve. Unlike transurethral resection of the prostate, it requires a fundamental shift in surgical approach to anatomic dissection along the surgical capsule rather than tissue resection [4]. This technique requires refined skills in endoscopic orientation, energy control, and hemostasis. Studies indicate that surgical proficiency requires a substantial number of cases, with learning curves varying by experience level [5].

Holmium:YAG laser has been the platform most widely used for anatomic endoscopic enucleation of the prostate (EEP), particularly for holmium LEP (HoLEP), which is well validated in the literature [6]. In recent years, thulium:YAG laser systems—available in both pulsed and continuous-wave configurations—have gained traction owing to their favorable tissue interaction characteristics, enhanced cutting precision, lower retropulsion, and superior hemostatic properties [7], [8].

As more urology centers transition between platforms, a key question arises: Does a switch in laser system impact surgical performance, outcomes, or complication rates? While theoretical benefits exist, current evidence remains inconclusive regarding the superiority of one laser over another [9], [10], [11]. Rather than focusing on head-to-head comparisons, it may be more relevant to assess how such transitions affect procedural efficiency and safety in real-world settings.

In this study, we evaluated the transition from HoLEP to thulium LEP (ThuLEP) with a pulsed Tm:YAG laser in a single institution, performed by surgeons with extensive HoLEP experience. We analyzed operative efficiency, energy use, and complication rates to determine whether the change in technology influenced surgical performance. By focusing on consecutive cases during the transition, our aim was to assess whether existing LEP expertise is directly transferable or if procedural adaptations are required.

## 2 Patients and methods

In this retrospective, single-center observational study we analyzed prospectively collected data for all patients undergoing EEP for symptomatic BPH between September 2015 and May 2025 at a high-volume tertiary care center.

Before adoption of thulium laser technology, 1725 HoLEP procedures had been performed by six surgeons, of whom three accounted for 88% of cases (820, 470, and 226 each). Following the institutional switch to ThuLEP, these three surgeons performed 1172 consecutive cases (583, 306, and 283 each) using the same EEP technique and workflow. The transition was implemented without formal retraining, with the assumption that HoLEP expertise would ensure safe and efficient adaptation to the new laser system.

Patients were included unless the preoperative prostate volume was <15 g to ensure meaningful analysis of efficiency metrics. No other exclusion criteria were applied, including prior prostate surgery, concurrent bladder procedures, or prostate cancer, to reflect real-world clinical practice.

A standardized en bloc EEP technique using the “three horseshoe incision” approach was applied in the majority of cases [12]. This method was used consistently throughout the ThuLEP phase and during most of the HoLEP phase, except for the initial HoLEP cases, for which the traditional three-lobe technique described by Gilling was used during the period of technique adaptation [13]. A Ho:YAG laser (Sphinx 100 W, LISA Laser, Katlenburg-Lindau, Germany; 550-μm fiber, 80 W, 2.5 J, 32 Hz) was used for HoLEP. For ThuLEP, a pulsed solid-state Tm:YAG laser (Thulio, Dornier MedTech, Weßling, Germany; 600-μm fiber, 100 W, 2.0 J, 50 Hz) was used.

Data were extracted from a prospectively maintained database and included baseline demographics (age, prostate-specific antigen [PSA], prostate volume, American Society of Anesthesiologists [ASA] score), urinary catheter status, anticoagulation/antiplatelet (ACAP) use, preoperative uroflowmetry, and postvoid residual urine volume (PVR).

Primary outcomes were postoperative complications within 30 d, including urinary retention, fever, clot evacuation, and transurethral coagulation, graded according to the Clavien-Dindo scheme. Procedural efficiency was assessed as the enucleation ratio efficiency (enucleated tissue/prostate volume), laser-to-prostate ratio (J/g tissue), and enucleation, morcellation, and overall operative efficiency (g/min).

Informed consent was waived and the study protocol was approved by the ethics committee of Albert-Ludwigs University Freiburg. The study was conducted in accordance with the principles of the Declaration of Helsinki.

### 2.1 Statistical analysis

Descriptive statistics are reported as the mean ± standard deviation for continuous variables. Group comparisons used nonparametric tests according to the data distribution. Segmented regression analysis was applied to assess changes in performance metrics over time, with modeling of both the slope and intercept across the HoLEP to ThuLEP transition. To account for potential baseline imbalances between groups, segmented regression models were adjusted for ASA score, urinary catheter status, and ACAP use. A *p* value <0.05 was considered statistically significant. Analyses were performed using Python version 3.11 (Python Software Foundation, Delaware, DE, USA).

## 3 Results

A total of 1516 HoLEP and 1172 ThuLEP procedures were analyzed. Baseline characteristics were largely comparable between the cohorts. There were no significant differences in patient age (70.65 ± 8.38 vs 70.43 ± 7.9 yr; *p* = 0.29), PSA (7.54 ± 10.43 vs 7.45 ± 11.09 ng/ml; *p* = 0.67), symptom severity (IPSS 19.91 ± 7.55 vs 19.35 ± 6.42; *p* = 0.08), quality-of-life score (3.48 ± 1.43 vs. 3.41 ± 1.22; *p* = 0.67), maximum urinary flow (Qmax; 11.22 ± 5.37 vs 11.41 ± 6.64 ml/s; *p* = 0.87), or PVR (119.6 ± 132 vs 132.3 ± 161.6 ml; *p* = 0.2302). Prostate volume was slightly higher in the ThuLEP group (102.3 ± 181.6 vs 93.2 ± 48.9 ml; *p* = 0.0545). The mean ASA score was significantly higher in the ThuLEP cohort (2.40 ± 0.63 vs 2.26 ± 0.63; *p* < 0.0001), which suggests modestly higher perioperative risk. An indwelling catheter (32.1% vs 25.1%; *p* = 0.0001) and ACAP use (28.2% vs 14.5%; *p* < 0.0001) were more prevalent in the HoLEP group. The prevalence of prostate cancer before EEP was similar in the two groups (3.7% vs 4.0%; *p* = 0.797; Table 1).

**Table 1:** Baseline characteristics of patients in the Ho:YAG and thulium:YAG groups for laser enucleation of the prostate

Parameter ^a^	Ho:YAG (*n* = 1516)	Thulium:YAG (*n* = 1172)	*p* value
Median age (yr)	70.65 ± 8.38	70.43 ± 7.9	0.29
Prostate-specific antigen (ng/ml)	7.54 ± 10.43	7.45 ± 11.09	0.67
International Prostate Symptom Score	19.91 ± 7.55	19.35 ± 6.42	0.08
Quality-of-life score	3.48 ± 1.43	3.41 ± 1.22	0.67
Maximum flow rate (ml/s)	11.22 ± 5.37	11.41 ± 6.64	0.87
Postvoid residual volume (m)	119.6 ± 132	132.3 ± 161.6	0.23
Prostate volume (ml)	93.2 ± 48.9	102.3 ± 181.6	0.05
American Society of Anesthesiologists score	2.26 ± 0.63	2.40 ± 0.63	<0.01
Indwelling catheter, *n* (%)	487 (32.1)	294 (25.1)	<0.01
Anticoagulant/antiplatelet medication, *n* (%)	429 (28.2)	170 (14.5)	<0.01
Prostate cancer, *n* (%)	61 (4)	44 (3.7)	0.79

Results are presented as mean ± standard deviation for continuous variables.

**Fig. 1:**
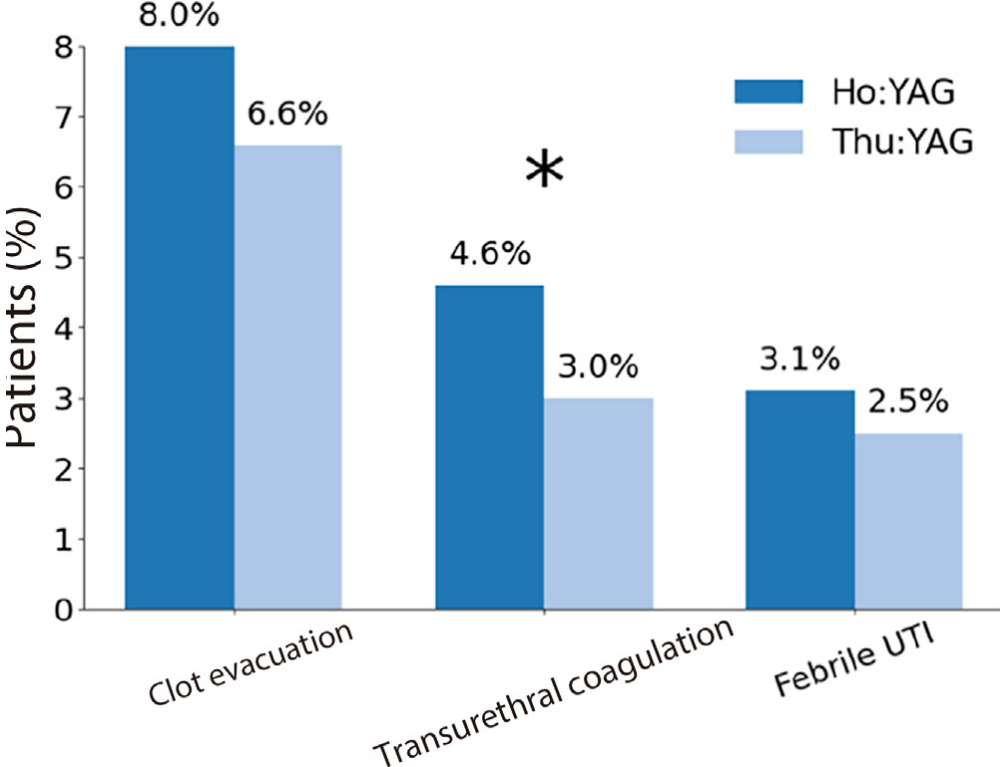
Perioperative complication rates in the Ho:YAG and thulium:YAG (Thu:YAG) groups for laser enucleation of the prostate. The rate of transurethral coagulation was significantly lower in the Thu:YAG group (* p < 0.05), while differences in clot evacuation and febrile urinary tract infection (UTI) were not statistically significant.

Complication rates were low in both groups, but with some notable differences. Postoperative urinary retention (grade I) was less frequent in the ThuLEP group (5.6% vs 9.3%; *p* = 0.0005), as was transurethral coagulation for bleeding (grade IIIb; 3.0% vs 4.6%; *p* = 0.0471). There was no significant difference in the incidence of clot evacuation (grade IIIa; *p* = 0.1971) or febrile urinary tract infection (grade II; *p* = 0.47; Fig. 1).

Efficiency metrics revealed better enucleation efficiency with thulium (1.64 ± 1.03 vs. 0.93 ± 0.49 g/min; *p* < 0.001), accompanied by greater energy use (1051.7 ± 1003.3 vs 898.2 ± 692.1 J/g; *p* < 0.001). The enucleation ratio efficiency was slightly lower for the ThuLEP group (0.71 ± 0.77 vs 0.77 ± 0.34; *p* < 0.001).

Segmented regression analysis (adjusted for ASA score, ACAP use, and urinary catheterization) revealed nuanced performance trends. The urinary retention rate declined during the HoLEP phase (*p* = 0.001), dropped further at the transition to ThuLEP (*p* = 0.033), and then gradually increased (*p* = 0.016). The transurethral coagulation rate also declined significantly during the HoLEP phase (*p* < 0.001), but showed no additional change on transition to ThuLEP or thereafter (*p* > 0.25), which suggests ongoing improvement independent of the laser type. For other complications, including clot evacuation and febrile urinary tract infection, no significant shifts in slope or intercept across the transition point were observed.

**Fig. 2:**
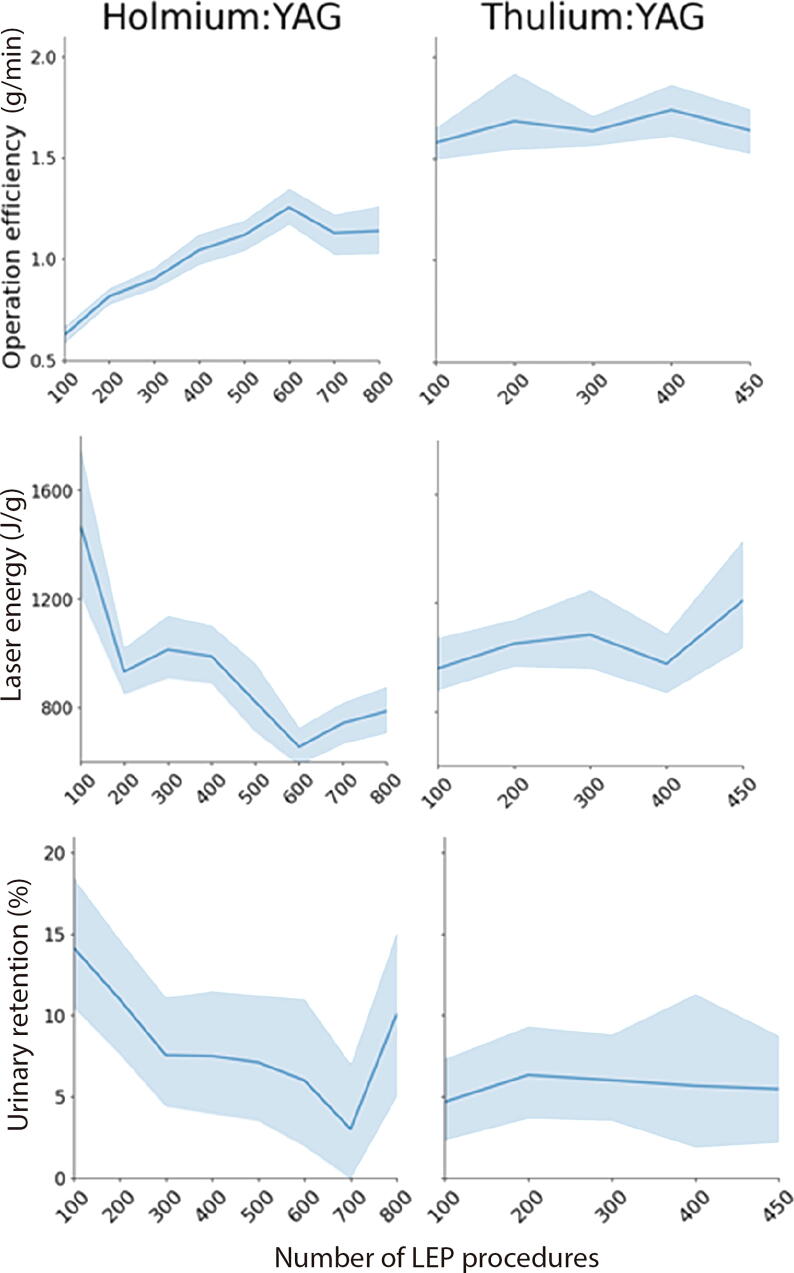
Trends in operative efficiency, laser energy use, and urinary retention in the transition from Ho:YAG to thulium:YAG for laser enucleation of the prostate (LEP), shown as line plots with 95% confidence interval. A marked increase in operative efficiency and a continuous decline in postoperative urinary retention rates were observed following thulium:YAG adoption. By contrast, the laser energy applied increased during this transition period.

Continuous intraoperative metrics revealed that energy use per gram decreased over time with HoLEP (*p* < 0.001), but increased again with ThuLEP (*p* < 0.001), which probably reflects adaptation to altered laser-tissue interaction. Operative efficiency improved before the transition (*p* < 0.001), jumped at the transition (*p* < 0.001), and then declined modestly (*p* < 0.001). Enucleation time mirrored this pattern, while morcellation time remained stable across all phases. No significant shift in enucleated tissue-to-volume ratio was observed on transition (*p* = 0.787), although a subtle decline occurred with ThuLEP (*p* = 0.001), which possibly indicates a more conservative resection approach (Fig. 2).

Taken together, these findings suggest that the laser switch induced temporary but manageable adjustments in surgical behavior, without compromising overall safety or long-term performance.

## 4 Discussion

LEP, particularly with Ho:YAG laser, has become the gold standard for surgical management of BPH. In recent years, thulium-based laser systems—including pulsed Tm:YAG—have gained momentum as promising alternatives. This shift has raised practical questions for surgeons transitioning from HoLEP to ThuLEP, including concerns about procedural adaptation, learning curves, and potential variations in outcomes. At our institution, a complete transition from HoLEP to the newly introduced pulsed solid-state Tm:YAG laser system was undertaken in 2022 [14]. We evaluated the impact of that transition, and the results demonstrated that ThuLEP is a safe and effective technique that allows anatomic EEP with high levels of postoperative patient satisfaction.

In our cohort, ThuLEP was associated with significantly lower incidence of postoperative urinary retention and transurethral coagulation, despite a modestly higher ASA risk profile. Enucleation efficiency was substantially greater under thulium, albeit at the cost of higher energy consumption per gram of tissue, while the enucleation ratio efficiency was slightly lower with ThuLEP. Although baseline characteristics were largely comparable, several relevant differences warrant consideration. The significantly higher mean ASA score in the ThuLEP group indicates a patient population with greater comorbidity and anesthetic risk, which could theoretically predispose to a higher rate of perioperative complications. By contrast, smaller proportions of patients in the ThuLEP group had an indwelling catheter or were on ACAP therapy than in the HoLEP cohort. It is known that both factors complicate LEP by impairing intraoperative visualization, prolonging hemostasis, and increasing the risk of postoperative bleeding or clot retention [15]. Likewise, chronic catheterization is often associated with bladder wall inflammation, which can potentially affect perioperative bladder recovery [16]. These differences should be taken into account when interpreting the outcome variations observed between the two groups*.*

These findings align with prior studies in large prostate cohorts. For a group of patients with a prostate size >80 ml, Zhang et al. [17] reported shorter operative and enucleation times for continuous-wave ThuLEP in comparison to HoLEP, while morcellation time and hospital stay were comparable. Similarly, Xiao et al. [18] found that continuous-wave thulium lasers achieved shorter enucleation times and better early functional outcomes, with no significant differences in complications. By contrast, a meta-analysis of randomized controlled trials (RCTs) by Hartung et al. [19] revealed no significant differences in operative time between the two modalities. However, the authors reported a significantly smaller decrease in hemoglobin and a lower rate of transient incontinence with ThuLEP, which suggests better intraoperative hemostasis. In our cohort, we observed a statistically significant reduction in transurethral coagulation rates with ThuLEP, although the absolute difference was modest and its clinical relevance remains uncertain. Nonetheless, these findings are directionally consistent with previous evidence indicating better hemostatic properties of thulium-based systems. However, these results are not directly comparable to ours, as the authors used a continuous-wave thulium laser. Nevertheless, continuous-wave and pulsed Tm:YAG systems share similar physical properties and laser-tissue interaction characteristics, which allows at least a contextual comparison of procedural trends and outcomes. Other comparative studies, including those by Shoma et al. [20] and Aybal et al. [21], confirm that both techniques offer excellent safety and functional outcomes, with no significant differences in complication profiles or urinary incontinence over time. However, these results are hardly comparable to ours, as the authors used a continuous-wave thulium laser.

While comparative data for HoLEP versus ThuLEP are informative, it is important to emphasize that our study differs fundamentally from classic RCTs. Unlike RCTs, the transition in our institution occurred after substantial experience had been gained in LEP, specifically with HoLEP. The three surgeons had collectively performed >1500 HoLEP procedures before adoption of thulium laser technology, which represents a level of procedural proficiency that probably influenced both efficiency and complication profiles. This experience advantage must be considered when interpreting the differences observed, particularly the lower rates of urinary retention and coagulation interventions in the ThuLEP group. The fact that several RCTs and meta-analyses failed to detect significant differences in these outcomes may, at least in part, reflect the influence of earlier stages of the learning curve in the study populations. Notably, it has been shown that even highly experienced HoLEP surgeons continue to improve over time. In a long-term series, Wenk et al. [22] demonstrated ongoing performance gains across 500 HoLEP cases, particularly in energy efficiency and enucleation speed, which demonstrates that technical refinement continues even after initial competency thresholds have been reached.

Our segmented regression analysis further supports this dynamic: enucleation efficiency improved markedly at the time of laser transition, but a subsequent slight decline over time suggests an adaptation phase to the new laser-tissue interaction profile. Similarly, energy consumption, which had decreased consistently under HoLEP, began to rise again after switching to ThuLEP, which probably reflects changes in energy delivery mechanics and surgical behavior during the transition. These findings echo those of Himmler et al. [23], who studied the reverse transition from ThuLEP to HoLEP. Although the two surgeons in their study both demonstrated high enucleation efficiency from the outset, one surgeon exhibited a measurable learning curve in laser energy efficiency, despite extensive prior experience. Importantly, complication rates and hemoglobin loss remained low and stable, which suggest that core anatomic enucleation skills are transferable, while device-specific metrics may still evolve during early adaptation.

Although anatomic EEP follows the same surgical principles regardless of the energy source, the physical characteristics of different laser systems can influence the surgeon’s dissection strategy, tissue handling, and intraoperative behavior. This is particularly relevant when comparing Ho:YAG and Tm:YAG platforms, which differ substantially in their laser-tissue interaction profiles. Thulium lasers emit energy at a slightly shorter wavelength (2013 nm vs 2120 nm for Ho:YAG) with higher water absorption and shallower tissue penetration, which results in precise vaporization with minimal collateral damage. The more continuous and homogeneous energy delivery of the Tm:YAG laser allows smoother dissection along the capsule and better coagulation at the point of contact. This in turn probably enhances endoscopic visibility, shortens the coagulation time, and reduces the need for secondary hemostasis interventions. These characteristics may explain the improvements in enucleation efficiency and the reduction in postoperative urinary retention and coagulation procedures observed following the switch from Ho:YAG to Tm:YAG. Similar benefits were reported by Zhang et al. [17], who found that continuous-wave thulium lasers achieved comparable enucleation results with less intraoperative blood loss. In a meta-analysis, Xiao et al. [18] noted better early postoperative Qmax and PVR values with thulium-based systems, which supports their potential advantages in tissue handling and early recovery.

However, it must be acknowledged that the improvements in enucleation efficiency and hemostatic control observed coincided not only with a change in laser medium and wavelength but also with greater nominal power (from 80 W to 100 W) and pulse frequency (from 32 Hz to 50 Hz). While these input parameters may have contributed to the effects seen, the distinct physical properties of the pulsed Tm:YAG laser, such as higher water absorption and lower penetration depth, are likely to be the principal drivers of the better surgical dynamics. Nonetheless, we recognize that this confounding factor limits causal attribution, and future studies with standardized power settings across platforms are warranted.

The higher energy consumption per gram of tissue observed with Tm:YAG may reflect a trade-off between precision and cumulative energy exposure. As highlighted by Bozzini et al. [24], greater irradiation time and total energy delivered during ThuLEP can theoretically intensify thermal effects, and could potentially contribute to postoperative irritative symptoms and transient erectile dysfunction. Although these effects were not observed in our cohort, we did not systematically assess postoperative erectile function or irritative symptoms, and therefore short-term functional consequences of higher cumulative energy cannot be fully excluded. This issue may also extend beyond short-term effects. In a recent study involving patients undergoing HoLEP at our institution, we observed that laser energy per gram of tissue (ie, laser efficiency) was significantly higher in the group of patients who later developed bladder neck stricture, which suggests that greater thermal exposure may contribute to local fibrosis or delayed healing responses [25]. Long-term outcomes were not systematically assessed in our study, but caution may be warranted in future studies using higher-energy pulsed laser modalities. Although operative efficiency was significantly higher with the pulsed Tm:YAG system, this was accompanied by a modest but statistically significant reduction in enucleation ratio efficiency. This suggests that faster enucleation may have been slightly more conservative in terms of adenoma volume removed. While the differences were not large, they highlight a potential trade-off between procedural speed and resection completeness that may be relevant for long-term outcomes in selected patients.

These considerations underscore the importance of optimizing energy modulation to balance tissue precision with thermal safety. Ultimately, while the choice of laser platform does not determine LEP success, it can modulate the surgical experience and influence tactical decisions—such as incision depth, capsule tracking, and energy modulation—and thereby affect procedural dynamics and perioperative outcomes.

Our study has several limitations. First, its retrospective and single-center design limits the generalizability of the findings to other institutions or health care systems. Second, the ThuLEP procedures after transition were performed exclusively by three high-volume surgeons with extensive prior HoLEP experience. This may have attenuated the observable learning curve and potentially overestimated the ease of transition for less experienced surgeons. Third, no structured functional follow-up was conducted, and outcome data such as postoperative IPSS, uroflowmetry, and patient-reported quality of life were not analyzed. Therefore, conclusions regarding long-term functional efficacy and patient satisfaction remain limited.

## 5 Conclusions

A transition from HoLEP to ThuLEP with a pulsed Tm:YAG laser is safe and effective in experienced hands. In our high-volume setting, the switch was associated with an immediate increase in enucleation efficiency, albeit at the cost of higher energy delivery per gram of enucleated tissue. Importantly, this was not accompanied by an increase in complication rates, which underscores the procedural safety and clinical viability of ThuLEP following HoLEP experience.

  ***Author contributions***: Maximilian Glienke had full access to all the data in the study and takes responsibility for the integrity of the data and the accuracy of the data analysis.

  *Study concept and design*: Glienke, von Bargen, Miernik.

*Acquisition of data*: Glienke, von Bargen, Özkan.

*Analysis and interpretation of data*: Glienke.

*Drafting of the manuscript*: Glienke.

*Critical revision of the manuscript for important intellectual content*: Miernik, Schönthaler, Wilhelm, Özkan.

*Statistical analysis*: Glienke.

*Obtaining funding*: None.

*Administrative, technical, or material support*: None.

*Supervision*: Gratzke, Miernik.

*Other*: None.

  ***Financial disclosures*:** Maximilian Glienke certifies that all conflicts of interest, including specific financial interests and relationships and affiliations relevant to the subject matter or materials discussed in the manuscript (eg, employment/affiliation, grants or funding, consultancies, honoraria, stock ownership or options, expert testimony, royalties, or patents filed, received, or pending), are the following: Arkadiusz Miernik reports research funds from the German Federal Ministry of Education and Research; travel support from the European Association of Urology and the German Society of Urology; consultant roles for KLS Martin, Avateramedical, LISA Laser, Schoelly fiberoptics, Dornier MedTech Systems, Medi-Tate, and B. Braun New Ventures; speaker roles for Richard Wolf and Boston Scientific; expert activities for Ludwig Boltzmann Gesellschaft; and interests in numerous medical technology patents and inventions. Christian Gratzke reports advisory roles for Astellas, Ipsen, Steba Biotech, Bayer, Olympus Winter & Ibe, Medi-Tate, MSD, AstraZeneca, and Roche; and speaker fees from Amgen, Astellas, Ipsen, Janssen-Cilag, Bayer, Takeda Pharmaceuticals, and medac. The remaining authors have nothing to disclose.

  ***Funding/Support and role of the sponsor*:** None.

  ***Use of generative AI and AI-assisted technologies***: ChatGPT-4.0 (OpenAI) was used to improve the clarity and readability of the manuscript. After using this tool, the authors reviewed and edited the content as needed and take full responsibility for the content of the publication.

  ***Data sharing statement***: The data generated and analyzed for this study are not publicly available but can be obtained from the corresponding author on reasonable request.

  ***Acknowledgments***: The authors acknowledge support via the Open Access Publication Fund of the University of Freiburg.
